# Validation of reference genes for whole blood gene expression analysis in cord blood of preterm and full-term neonates and peripheral blood of healthy adults

**DOI:** 10.1186/s12864-021-07801-0

**Published:** 2021-06-30

**Authors:** Kristin Hieronymus, Benjamin Dorschner, Felix Schulze, Neeta L. Vora, Joel S. Parker, Jennifer Lucia Winkler, Angela Rösen-Wolff, Stefan Winkler

**Affiliations:** 1 Department of Pediatrics, University Hospital Carl Gustav Carus, Technische Universität Dresden, Fetscherstraße 74, 01307 Dresden, Germany; 2 Department of Obstetrics and Gynecology, University of North Carolina School of Medicine, Chapel Hill, North Carolina USA; 3 Department of Genetics, Lineberger Comprehensive Cancer Center, University of North Carolina, Chapel Hill, North Carolina USA; 4 Department of Gynecology and Obstetrics, University Hospital Carl Gustav Carus, Technische Universität Dresden, Dresden, Germany

**Keywords:** Housekeeping genes, Comparative gene expression analysis, Validation, Whole blood, Neonates, Infants

## Abstract

**Background:**

Preterm birth is the leading cause of neonatal morbidity and mortality, but research efforts in neonatology are complicated due to the unavailability of large volume blood samples. Whole blood assays can be used to overcome this problem by performing both functional and gene expression studies using small amounts of blood. Gene expression studies using RT-qPCR estimate mRNA-levels of target genes normalized to reference genes. The goal of this study was to identify and validate stable reference genes applicable to cord blood samples obtained from developing neonates of different gestational age groups as well as to adult peripheral blood samples. Eight reference gene candidates (*ACTB, B2M, GAPDH, GUSB, HPRT, PPIB, RPLP0, RPL13*) were analyzed using the three published software algorithms Bestkeeper, GeNorm and NormFinder.

**Results:**

A normalization factor consisting of *ACTB* and *PPIB* allows for comparative expression analyses of neonatal samples from different gestational age groups. Normalization factors consisting of *GAPDH* and *PPIB* or *ACTB* and *GAPDH* are suitable when samples from preterm and full-term neonates and adults are compared. However, all candidate reference genes except *RPLP0* exhibited significant intergroup gene expression variance and a higher gene expression towards an older age which resulted in a small but statistically significant systematic bias. Systematic analysis of RNA-seq data revealed new reference gene candidates with potentially superior stability.

**Conclusions:**

The current study identified suitable normalization factors and proposed the use of the additional single gene *RPLP0* to avoid systematic bias. This combination will enable comparative analyses not only between neonates of different gestational ages, but also between neonates and adults, as it facilitates more detailed investigations of developmental gene expression changes. The use of software algorithms did not prevent unintended systematic bias. This generally highlights the need for careful validation of such results to prevent false interpretation of potential age-dependent changes in gene expression. To identify the most stable reference genes in the future, RNA-seq based global approaches are recommended.

**Supplementary Information:**

The online version contains supplementary material available at 10.1186/s12864-021-07801-0.

## Background

The clinical course of preterm neonates is often complicated by acute or chronic diseases, such as necrotizing enterocolitis or bronchopulmonary dysplasia which are typically based on the immaturity of organs or the immune system. Although significant progress has been made in understanding these diseases, the unavailability of large volume blood samples required to isolate specific leukocyte fractions for functional or gene expression studies complicates research advances in neonatology. Recently, several studies using assays based on whole blood have been published [1–5]. As these assays do not require the isolation of specific leukocyte subsets, they are suitable for diagnostic and research purposes in neonates who can only donate small amounts of blood. Whole blood assays can be used to perform both functional and gene expression studies [4].

Quantitative real-time PCR (RT-qPCR) is a powerful tool to monitor changes in gene expression between different tissues or over time. In order to identify true biological variation, technical variation needs to be minimized. Apart from introducing proper standard procedures throughout the qPCR workflow, normalization using reference genes is recommended [6]. The expression of reference genes should be stable and independent of the experimental variable. In the past, genes like *ACTB* or *GAPDH* were often used as reference genes without further validation for the experimental setup, despite data proving their variability in certain tissues or under certain conditions [7]. It is crucial to validate reference genes with regard to sample type and experimental setup. The use of a normalization factor based on geometric averaging of multiple validated reference genes instead of a single reference gene was shown to further reduce technical variation within the samples [8]. This strategy is still not sufficiently applied in experimental research [9, 10]. The algorithms BestKeeper, NormFinder and GeNorm were developed to enable a comparison of the stability of the candidate reference genes [8, 11, 12]. The BestKeeper and GeNorm algorithms are based on pairwise correlation of each reference gene with an optimal normalization factor merging data from all analyzed reference genes. The NormFinder algorithm calculates expression stability based on intra- and intergroup variation of each candidate reference gene.

The primary goal of this study was to identify and validate stable reference genes applicable to cord blood obtained from developing neonates of different gestational age groups. Since many whole blood RNA expression data stem from adult samples, the secondary goal of the study was to identify stable reference genes that facilitate the direct comparison of neonatal and adult expression data. Eight candidate reference genes (*ACTB, B2M, GAPDH, GUSB, HPRT, PPIB, RPLP0, RPL13*) were analyzed for their expression stability in cord blood of preterm neonates, full-term neonates, and peripheral blood of healthy adults.

## Results

### Patient characteristics & group assignments

A total of 30 patients were assigned to three groups based on their age: 10 preterm neonates (group 1), 10 full-term neonates (group 2), and 10 healthy adult controls (group 3). The mean gestational age was 30.6 weeks for group 1 and 38.9 weeks for group 2 (Table 1). Exposure to antenatal corticosteroids was higher in group 1. The mode of delivery was similar in both groups, with a high frequency of caesarean sections. According to local protocol, preterm neonates < 34 weeks of gestation were delivered via caesarean section. The indications for caesarean delivery of full-term neonates (group 2) were cephalopelvic disproportion (*n* = 1), previous caesarean section (n = 4), breech presentation (*n* = 1), and uterus myomatosus (*n* = 1). The reasons for preterm birth were spontaneous preterm labor with or without premature rupture of membranes (*n* = 6), intrauterine growth retardation associated with pathological cardiotocography or pathological doppler findings (n = 2), and pre-eclampsia or hemolysis, elevated liver enzymes, low platelet count (HELLP) syndrome (*n* = 2). None of the neonates in the study developed bacterial infection within 72 h after birth.

The mean age of group 3 was 29.3 years (range 21–46). The participants did not show any clinical signs of infectious disease, did not suffer from acute or chronic diseases, and were not being treated with any long-term medication.

**Table 1:** Maternal and newborn characteristics by group

Measure	Preterm neonates (group 1, *n* = 10)	Full-term neonates (group 2, *n* = 10)	*p*-value
Maternal age, mean (range), years	30 (23–36)	32.3 (23–39)	0.289
Receipt of antenatal corticosteroids			0.003
* None, n (%)*	4 (40)	10 (100)	
* Incomplete course, n (%)*	0 (0)	0 (0)	
* Full course, n (%)*	6 (60)	0 (0)	
Mode of delivery			> 0.999
* Caesarean deliveries, n (%)*	7 (70)	7 (70)	
* Spontaneous deliveries, n (%)*	3 (30)	3 (30)	
Gestational age, mean (range), weeks	30.6 (23.4–35.9)	38.9 (37.3–40.4)	< 0.0001
Birth weight, mean (range), g	1419 (465–2260)	3313 (2500–4570)	< 0.0001
< 10th centile birth weight, n (%)	3 (30)	1 (10)	0.264
Umbilical artery pH, mean (range)	7.31 (7.13–7.43)	7.31 (7.22–7.37)	0.954
Infant sex			0.361
* Female, n (%)*	5 (50)	7 (70)	
* Male, n (%)*	5 (50)	3 (30)	

### Selection of genes for normalization & validation strategy

**Fig. 1:**
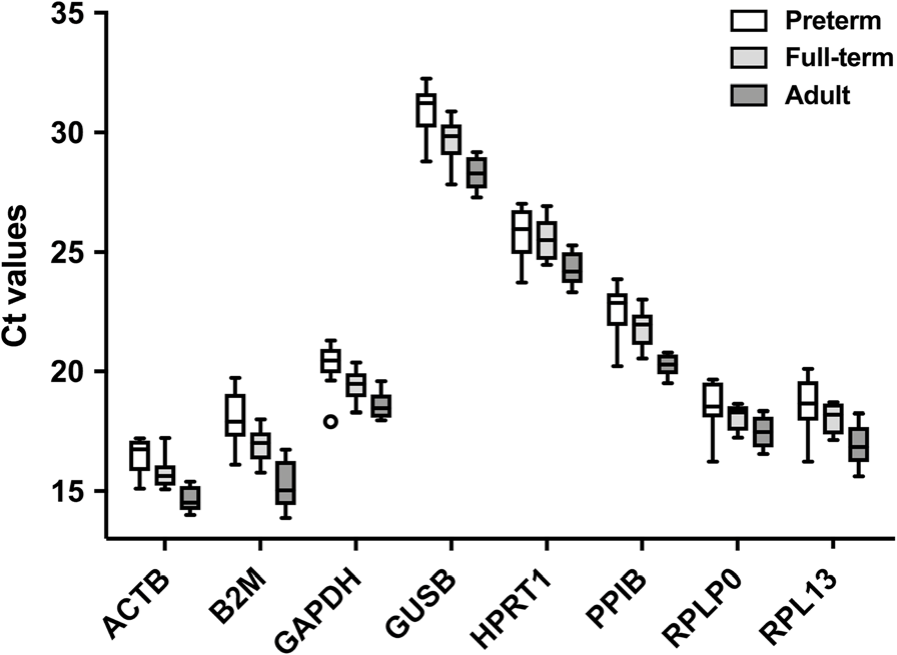
RT-qPCR cycle threshold values of candidate reference genes differ between experimental groups. Cycle threshold values (ct) are depicted as Tukey boxplot (plotted are median, inter-quartile-range (IQR) (box), 75th percentile + 1.5 IQR or 25th percentile – 1.5 IQR (whiskers) and outliers). *n* = 10 per group

Based on a literature review eight commonly used reference genes *ACTB*, *B2M*, *GAPDH*, *GUSB*, *HPRT*, *PPIB*, *RPLP0* and *RPL13* were chosen and evaluated for their expression stability in cord blood of preterm neonates, full-term neonates, and peripheral blood of healthy adults (Table 2). The mean cycle threshold (ct) values for the transcripts ranged between 15.6 and 29.6 cycles, indicating high to medium expression. The highest expression was found for *ACTB*, while *GUSB* was the least expressed (Fig. 1). Ct values correlated inversely with volunteers’ age, indicating differential gene expression between groups.

Three different, previously published algorithms were employed to assess reference gene stability: BestKeeper, NormFinder, and GeNorm [8, 11, 12]. A two-step validation strategy was chosen: For each algorithm, the expression stability of the candidate reference genes was analyzed for groups 1 and 2 (preterm and term neonates) in a first step to test for differences between different gestational ages. In a second step, the expression stability was analyzed for groups 1–3 together in order to compare neonatal and adult expression data directly.

**Table 2:** Name, function, primer sequences and amplicon characteristics of candidate reference genes (bp, base pairs; E, PCR efficiency)

Name	Accession No.	Function	Sequence (5‘ -> 3‘)	Amplicon (bp)	*E* (%)
*ACTB*	NM_001101	Cytoskeletal structural protein	F: AGA GCT ACG AGC TGC CTG AC R: AGC ACT GTG TTG GCG TAC AG	184	92.48
*B2M*	NM_004048	Beta-chain of MHC-I molecules	F: CAC TGA ATT CAC CCC CAC TGA R: CTG CTT ACA TGT CTC GAT CCC A	104	92.94
*GAPDH*	NM_002046	Enzyme of glycolysis	F: CCA TGA GAA GTA TGA CAA CAG CC R: GGG TGC TAA GCA GTT GGT G	70	96.28
*GUSB*	NM_000181	Exoglycosidase in lysosomes	F: ACT TCT CTG ACA ACC GAC GC R: AGG ATC ACC TCC CGT TCG TA	172	95.21
*HPRT*	NM_000194	Glycosyltransferase mutase	F: GAC CAG TCA ACA GGG GAC AT R: AAG CTT GCG ACC TTG ACC AT	167	93.83
*PPIB*	NM_000942	Cyclosporine-binding protein	F: GCC GGG TGA TCT TTG GTC TC R: AAG TCT CCG CCC TGG ATC AT	148	100.55
*RPLP0*	NM_001002	Ribosomal protein	F: TGG CAG CAT CTA CAA CCC TG R: ATC TGC AGA CAG ACA CTG GC	102	90.21
*RPL13*	NM_012423	Ribosomal protein	F: CGA GGT TGG CTG GAA GTA CC R: CTT CTC GGC CTG TTT CCG TAG	121	92.44

### Ranking of expression stability of single reference genes based on published algorithms

**Fig. 2:**
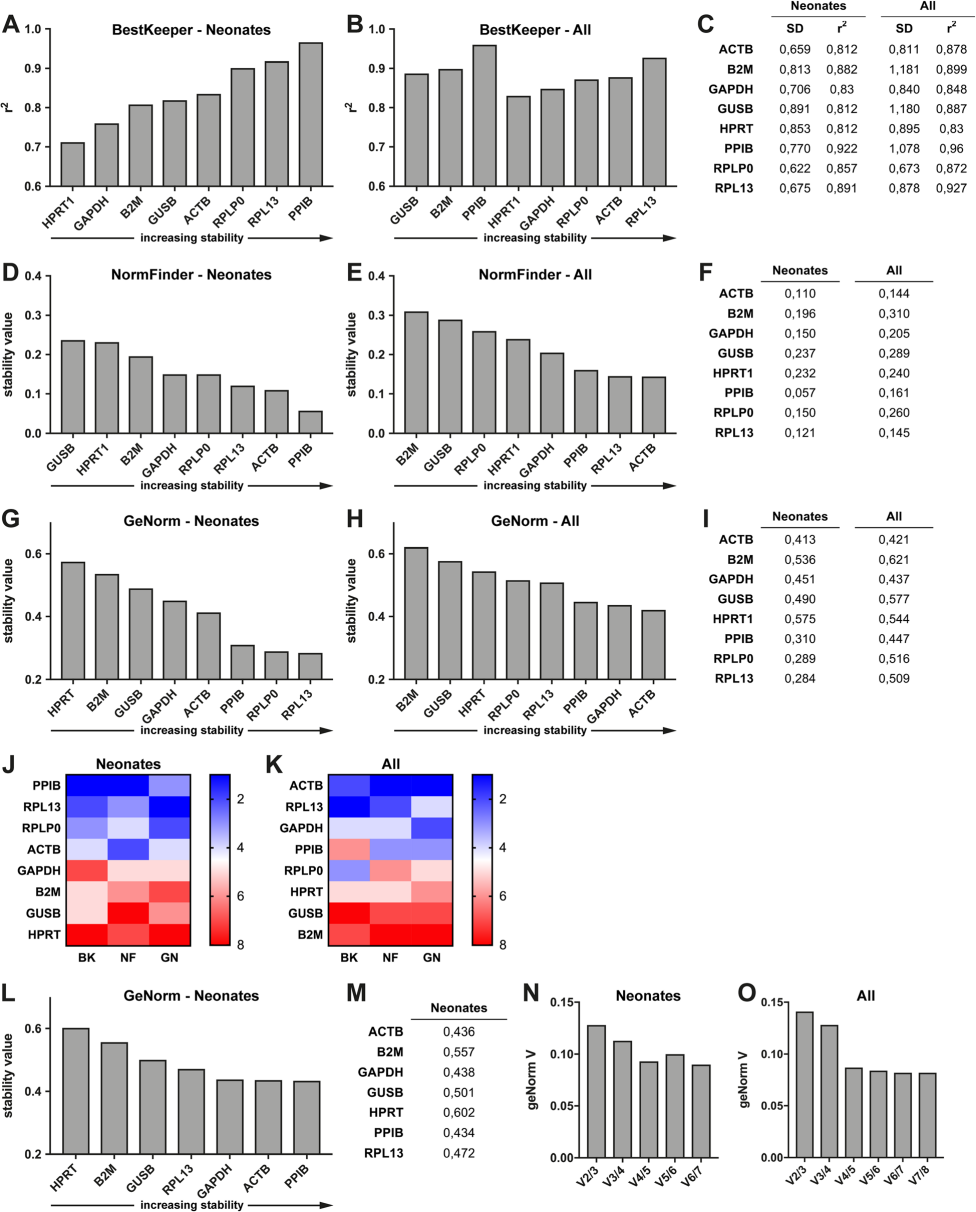
Algorithm based ranking of candidate reference genes. **a** – **b** BestKeeper based calculation of the coefficient of determination (r^2^) for groups 1 and 2 (neonates) or groups 1–3 (all). **c** Standard deviation and r^2^ for groups 1 and 2 (preterm and full-term neonates) or groups 1–3 (all). **d** – **f** NormFinder based calculation of stability values for groups 1 and 2 (preterm and full-term neonates) or groups 1–3 (all). **g** – **i** GeNorm based calculation of stability values for groups 1 and 2 (preterm and full-term neonates) or groups 1–3 (all). **j** – **k** Overall ranking of candidate reference genes for groups 1 and 2 (preterm and full-term neonates) or groups 1–3 (all). **l** – **m** GeNorm based calculation of stability values for groups 1 and 2 (preterm and full-term neonates) after exclusion of *RPLP0* from the analysis. **n** - **o** GeNorm based calculation of the number of reference genes needed to build a stable normalization factor for groups 1 and 2 (preterm and full-term neonates) or groups 1–3 (neonates and healthy adults). BK, BestKeeper; NF, NormFinder; GN, GeNorm

Using BestKeeper, the highest r^2^ values, corresponding to high expression stability, were calculated for *PPIB* and *RPL13* when comparing groups 1 and 2 (Fig. 2, a and c). *RPL13* and *ACTB* exhibited the highest r^2^ values (Fig. 2, b and c) when pooling data from groups 1 and 2 and comparing them to group 3. The NormFinder algorithm calculated the lowest stability values for *PPIB* and *ACTB*, which corresponded with the highest expression stability for groups 1 and 2. *ACTB* and *RPL13* offered the highest expression stability (Fig. 2, d – f) when groups 1, 2 and 3 were jointly analyzed. Based on the GeNorm calculations, *RPL13* and *RPLP0* offered the highest stability between groups 1 and 2. *ACTB* and *GAPDH* offered the highest stability when pooling groups 1 and 2 and comparing them to group 3 (Fig. 2, g - i).

The overall expression stability of each gene was assessed by the mean rank of all three calculations. The highest expression stability was achieved by *PPIB* between neonatal groups 1 and 2, and by *ACTB* when analyzing neonatal data from groups 1 and 2 together with the adult group 3 (Fig. 2, j and k).

### Calculation of a normalization factor based on multiple reference genes

Gene expression normalization using a normalization factor based on the geometric mean of multiple validated reference genes is recommended [13]. This calculation was supported by the algorithms NormFinder and GeNorm. When groups 1 and 2 were analyzed, *ACTB* and *PPIB* were selected as parts of the normalization factor by the NormFinder algorithm. As the expression stability of the resulting normalization factor ACTB/PPIB was lower than the best ranked gene *PPIB* alone (stability value 0.062 vs. 0.057), the use of this combination cannot be supported. When groups 1, 2, and 3 were analyzed together, *GAPDH* and *PPIB* were chosen for the normalization factor. The expression stability of the resulting normalization factor *GAPDH/PPIB* was higher than the best ranked gene *ACTB* alone (stability value 0.123 vs. 0.207).

Following GeNorm analysis for groups 1 and 2, *RPLP0* and *RPL13* were selected for the normalization factor. Because both genes represent ribosomal genes, *RPLP0* was removed from the GeNorm dataset and the analysis was repeated. The combination of *ACTB* and *PPIB* was then chosen as the normalization factor with V = 0.128 for neonatal samples of different gestational age (Fig. 2, l-n). When groups 1, 2, and 3 were analyzed together, the combined use of *ACTB* and *GAPDH* offered sufficient expression stability (V = 0.141) (Fig. 2, o).

### The correct choice of reference genes is important when assessing age-related expression changes

**Fig. 3:**
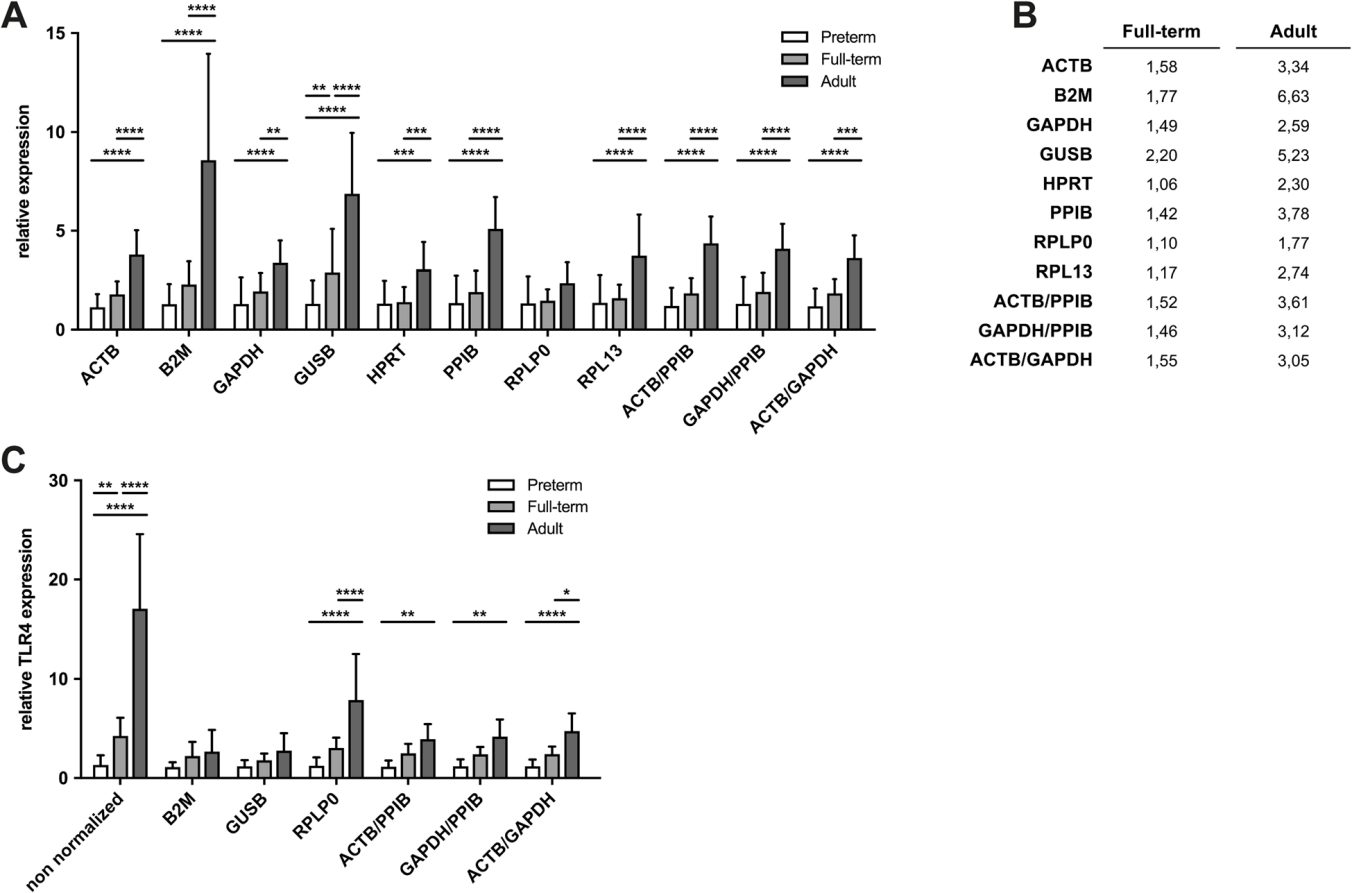
Reference genes introduce systematic bias despite algorithm based validation. **a** Relative gene expression (normalized to the mean of group 1 (preterm neonates)) for each candidate reference gene or normalization factor. **b** Fold-change difference in gene expression of full-term neonates or healthy adults relative to preterm neonates. **c** Relative gene expression of *TLR4* from cord blood of preterm or full-term neonates and peripheral blood of healthy adults is shown non-normalized or normalized to different candidate reference genes or normalization factors. **a**, **c** Data is presented as mean ± SD

Most reference gene candidates in this study exhibited an age-dependent increase in relative non-normalized gene expression (Fig. 3, a). The maximum fold-change between groups 1 and 2 was found for *GUSB*, while the maximum fold-change between groups 1 and 3 was detected for *B2M*. The lowest differences in gene expression between groups was found for *RPLP0*, offering a mean fold-change difference of 1.10 between groups 1 and 2, and 1.77 between groups 1 and 3 (Fig. 3, b). Overall, the expression patterns of reference genes were comparable between groups 1 and 2, but differed significantly when group 1 or 2 were compared to group 3 (neonates vs. adults). Despite being proposed by GeNorm and Normfinder, the normalization factors (*ACTB*/*PPIB, GAPDH*/*PPIB* and *ACTB*/*GAPDH*) also exhibited statistically significant differences of intergroup gene expression when group 1 or 2 were compared to group 3 (neonates vs. adults).

Analyzing the data with regard to the mode of delivery did not reveal differences in reference gene expression between neonates born via caesarean section and neonates born via spontaneous vaginal delivery (Fig. S1 a – d).

A higher expression of reference genes in one experimental group will underestimate normalized expression of target genes in this group, thereby contributing to systematic bias. *TLR4* was chosen as an example to illustrate this effect, since the expression of *TLR4* in monocytes is known to be age-dependent with an increase in gene expression towards adulthood [14]. As expected, a statistically significant increase of *TLR4* expression was observed in group 3 when compared to group 1 or 2 (Fig. 3, c). Normalization of *TLR4* gene expression using reference genes with strong intergroup gene expression variance like *GUSB* or *B2M* abrogated the differences in *TLR4* expression between the groups. Reference genes with lower inter-group expression difference or the proposed normalization factors preserved the result of the differential *TLR4* expression (Fig. 3, c).

### Candidate reference gene validation on existing RNA-seq datasets and systematic screening for further potential candidates

**Fig. 4:**
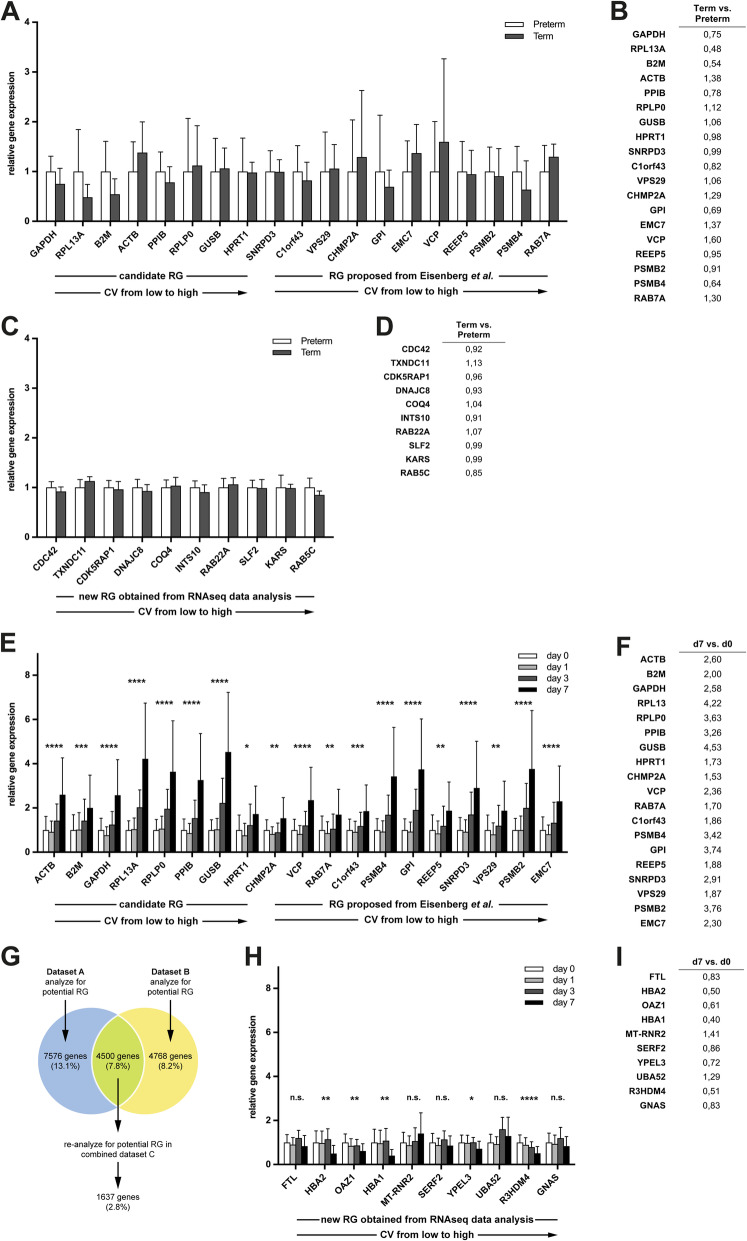
RNAseq data can be used to assess performance of candidate reference genes and to screen for new reference genes. **a** Relative expression of candidate reference genes based on the RNA-seq data from cord blood of preterm and full-term neonates [15]. Data was normalized to the group mean gene expression of preterm neonates and presented as mean ± SD. **b** Fold-change gene expression difference between full-term and preterm neonates. **c** Following systematic analysis of the RNA-seq data, the relative expression of genes with the lowest CV are depicted. Data was normalized to the group mean gene expression of preterm neonates and presented as mean ± SD. **d** Fold-change gene expression difference between full-term and preterm neonates. **e** Relative gene expression of candidate reference genes based on the RNA-seq data from peripheral blood of full-term neonates taken at day of life (DOL) 0 and additionally at either DOL 1, 3 or 7 [17]. Data was normalized to the group mean gene expression of DOL 0 and presented as mean ± SD. **f** Fold-change gene expression difference between DOL 7 and DOL 0. **g** Systematic analysis of RNA-seq datasets GSE123070 (dataset “A”) and GSE111404 (dataset “B”) [17]. **h** Following systematic analysis of the RNA-seq data, the relative expression of genes with the lowest CV are depicted. Data was normalized to the group mean gene expression of DOL 0 and presented as mean ± SD. **i** Fold-change gene expression difference between DOL 7 and DOL 0

To overcome the limits of a small sample size, the findings of the current study were validated in a dataset containing RNA-seq data of cord blood of preterm and full-term neonates (n = 8 for each group) previously published by Vora et al. [15]. The reference gene candidates *HPRT1* and *RPLP0* showed low fold-change differences between preterm and full-term neonates (Fig. 4, a and b). Next, we used this RNA-seq dataset to compare the performance of our set of candidate reference genes with reference genes proposed by the RNA-seq based study by Eisenberg et al. [16]. Overall, both sets of reference genes performed comparably (Fig. 4, a and b). In addition, the whole RNA-seq dataset published by Vora et al. was screened for further potential reference gene candidates. Systematic analysis retrieved 2401 potential reference genes. The 10 genes with the lowest coefficient of variation (CV) are shown in Fig. 4 (c and d). *CDC42* is a promising target with low intra- and intergroup variability.

To shed light on the question whether the reference genes under investigation in this study would perform equally well using both neonatal cord blood and neonatal peripheral blood, publicly available RNA-seq data from peripheral blood of full-term neonates [17] were analyzed. The dataset published by Lee et al. includes samples of full-term neonates at day of life (DOL) 0 and additionally at either DOL 1, 3, or 7 [17]. Again, the set of reference genes investigated in the currrent study and the one proposed by Eisenberg et al. performed comparably (Fig. 4, e and f) [16]. All candidate reference genes, however, exhibited increased gene expression with increasing postnatal age. A systematic analysis of the dataset published by Lee et al. for potential new reference genes with superior stability revealed 1637 potential reference genes (Fig. 4, g). The 10 genes with the lowest CV are shown in Fig. 4, (h and i). Although offering a low CV, several of these candidate reference genes still exhibit strong expression differences in neonatal peripheral blood between DOL 0 and 7 (Fig. 4, i). *FTL* is a promising candidate with low intra- and intergroup variability which may serve as a reference for gene expression analysis in the first days of life.

## Discussion

### Two sets of reference gene were identified and validated for age-related comparative expression analyses

Eight candidate reference genes were selected based on a literature review and systematically analyzed for their expression stability in cord blood of preterm neonates, full-term neonates and peripheral blood of healthy adults. Three different algorithms were employed to assess expression stability of reference genes in order to increase the robustness of our findings [18–20]. The combination of two reference genes, *ACTB* and *PPIB*, was identified as an optimized normalization factor when investigating developmental expression changes in preterm and full-term neonates (groups 1 and 2). For comparisons between neonatal and adult samples (groups 1–3), e.g. when investigating innate immune processes, *GAPDH* and *PPIB* or *ACTB* and *GAPDH* are suitable normalization factors.

The candidate reference gene expression patterns and proposed normalization factors were stable in neonatal cord blood of group 1 and 2 (preterm and full-term neonates) and may serve as standards for normalizing gene expression in future studies. The expression patterns were more complex when neonatal and adult groups are compared (groups 1 or 2 vs. 3). Almost all reference genes and all normalization factors exhibited an increased expression with small but statistically significant differences in adults compared to neonates, which may contribute to systematic bias. Despite these expression changes, the known age-dependent expression of TLR4 [14] was demonstrated using the proposed normalization factors. The low fold-change in expression of normalization factors between neonates and adults (groups 1 or 2 vs. 3) and the successful proof of age-dependent TLR4 expression support the interpretation that these differences are of marginal relevance. One gene, *RPLP0*, did not display statistically significant expression regulation between neonates and adults, but was dismissed due to its higher intragroup expression variation. The authors suggest using one of the proposed normalization factors and, in addition, *RPLP0* as a single reference gene. Together with the finding that *GAPDH* and *PPIB* are stable reference genes in comparative analyses of pediatric and adult patients [21, 22], this approach facilitates comparative gene expression analyses between human subjects of all age groups, and thereby provides the basis for a better understanding of developing immunity. The broad applicability of the proposed reference genes in this study is further supported by their successful validation in previously published RNA expression datasets.

There has been an ongoing controversy over the use of neonatal cord blood as a surrogate for neonatal peripheral blood. Based on immune cell composition and mass cytometry analyses, Olin et al. reported of strong differences between cord blood and peripheral blood of neonates [29], but the predictive value of neonatal cord blood remains unclear for gene expression analysis. Although the candidate reference genes of the current study performed sufficiently in cord blood from preterm and full-term neonates, applying them to RNA-seq data of peripheral blood from neonates revealed their dynamic regulation during the first week of life. Future research should address the question whether these differences are mainly caused by differences between cord blood and peripheral blood, or if they are induced by dynamic changes of the immune system during the first days of life [18, 29].

The selection of candidate reference genes in this work was guided by a literature-based approach. Benchmarking this set of reference genes with reference genes proposed by a study using a global tissue-based RNA-seq approach exhibited comparable stability [16]. Another strategy is the systematic screening of RNA-seq data of cord blood from preterm and full-term newborns to reveal new potential reference genes. Apart from low coefficients of variation, candidate reference genes derived by such an approach should not exhibit expression differences between samples of preterm and full-term neonates. Nevertheless, the candidate reference genes identified by this approach require validation in the specific experimental setup.

### Generalizability and implications of the reference genes validated by this study

Considering risk factors for preterm delivery and state-of-the-art treatment strategies, a variety of factors are known to influence fetal/neonatal gene expression, and have to be controlled for when investigating reference gene candidates: sex, fetal growth restriction, application of antenatal corticosteroids, the etiology of preterm delivery, and the mode of delivery itself [23–26]. Sex distribution, frequency of fetal growth restriction, and mode of delivery did not differ between both groups of neonates (groups 1 and 2), and are comparable to cohorts of clinical trials [27, 28]. Current study preterm birth causes were comparable to those in larger cohorts of preterm neonates: spontaneous preterm labor with or without premature rupture of membranes; maternal and fetal indications for delivery [29, 30]. Although this was only a one-center study, these group 1 and 2 neonates had patient characteristics similar to those reported in larger clinical trials, indicating that these results may be generalized.

Antenatal corticosteroid treatment is a key element in the setting of probable preterm delivery, significantly decreasing respiratory distress syndrome and mortality from premature birth. Apart from their known benefits, antenatal corticosteroids may also lead to adverse long-term health consequences by driving changes in gene regulation [24, 31]. Populations of preterm neonates included in clinical studies typically offer a high coverage of antenatal steroids, while full-term neonates do not [27, 28, 32]. This intergroup difference might induce experimental bias to expression of candidate reference genes. Saugstad and colleagues analyzed the impact of antenatal corticosteroids on the genome-wide gene expression in leukocytes of neonatal peripheral blood and found thirteen genes to be expressed differentially [24]. The reference gene set investigated in this study was not affected by antenatal corticosteroid treatment and is therefore ideal for the investigation of gene expression changes that depend on gestational age and medical intervention.

### Limitations

The current study has two limitations: (1) the small sample size of n = 10 per group, which may reduce the generalizability of the findings. Nevertheless, both groups of neonates exhibited typical clinical characteristics. (2) The selection of candidate reference genes was based on literature review. For future studies, RNA-seq based approaches will help select and validate superior candidates.

## Conclusions

This study identified normalization factors for gene expression studies in cord blood of preterm or full-term neonates and peripheral blood of healthy adults. Considering the age-dependent increase in reference gene expression, it seems reasonable to use *ACTB/PPIB* for the group of neonates and *GAPDH*/*PPIB* or *ACTB*/*GAPDH* for the group of neonates and adults. The additional use of *RPLP0* as a single reference gene is recommended to further reduce systematic bias. In the future, systematic analysis of RNA-seq data - including all pediatric age groups - will help identify an improved set of reference genes.

## Methods

### Patients

Inclusion criteria for neonates were inborn delivery and gestational age ≤ 36 + 6 weeks (group 1) or 37 + 0–42 + 0 weeks (group 2). Exclusion criteria for groups 1 and 2 were prolonged premature rupture of membranes > 24 h, signs of chorioamnionitis and neonatal asphyxia (umbilical artery pH < 7.0, 5-minute Apgar score ≤ 5). Inclusion criteria for the adult participants (group 3) were age ≥ 18 years. Exclusion criteria were any acute or chronic diseases and medical treatment. The study was approved by the responsible ethics committee (Ethics committee of the TU Dresden, EK97032014) before start.

### RNA isolation

Cord blood from neonates born preterm or full-term as well as peripheral blood from healthy adult volunteers was drawn into PAXgene Blood RNA tubes (PreAnalytix, 762,165) after obtaining written informed consent. Cord blood was drawn immediately after birth. Blood was stored at -20 °C until RNA isolation. Total RNA was purified using the PAXgene Blood RNA kit (PreAnalytix, 762,174) according to the manufacturer’s instructions including DNAseI digestion. RNA concentration was quantified using the Quantifluor RNA system (Promega GmbH, Mannheim, Germany) and the Quantus Fluorometer.

### cDNA synthesis and RT-qPCR

cDNA synthesis was performed using M-MLV reverse transcriptase (Promega GmbH, Mannheim, Germany). 1 µg RNA was mixed with 0.5µL random hexamer (10µM) and 0.5µL oligo dT primer (10µM) and filled up with H_2_O to 25.4µL reaction volume. After incubation at 70 °C for 10 min and chilling at 4 °C for 5 min, the reverse transcriptase mixture including buffer, dNTPs, RNAsin Plus (Promega GmbH, Mannheim, Germany) and reverse transcriptase was added (complete reaction volume 35µL). Transcription was performed at 42 °C for 60 min and inactivated at 95 °C for 5 min. The cDNA obtained was stored at -20 °C until use.

For RT-qPCR, GoTaq qPCR Master Mix (Promega GmbH, Mannheim, Germany) was used according to the manufacturer’s instructions with primer concentrations of 0.25µM. RT-qPCR was run on the Applied Biosystems 7300 Real-Time PCR System in triplicates. PCR conditions included an initial linearization step at 95 °C for 10 min, followed by 40 cycles of denaturation at 95 °C for 15 s and annealing/extension at 60 °C for 1 min. Afterwards, the melting curve was analyzed to determine specificity of reaction products.

### Gene selection and primer design

Eight genes from various functional classes commonly used as reference genes were selected (*ACTB, B2M, GAPDH, GUSB, HPRT, PPIB, RPLP0, RPL13)*. Primers were designed using Primer3 software via PrimerBlast (NCBI) and selected to produce amplicons spanning two exons. Specificity was validated using cDNA from whole blood in endpoint PCR assays. PCR products were separated on a 2.5 % agarose gel to validate expected size.

### Software algorithms to assess expression stability

Expression stability of selected reference genes was analyzed using BestKeeper [11], NormFinder [12] or GeNorm algorithms (qbase + software V3.1, (Biogazelle, Zwijnaarde, Belgium)) [8].

The BestKeeper algorithm is based on (a) expression stability of the genes measured via the standard deviation (SD) of the ct values and (b) the correlation between each candidate reference gene and the BestKeeper Index measured by the coefficient of determination (r^2^). Using this algorithm, genes with a low standard deviation and a high coefficient of determination are ranked as the most stable reference genes. Genes with a SD > 1, indicating starting template variation of a factor greater than 2, were ranked at the lowest position and the remaining genes were ordered based on their coefficient of determination. BestKeeper software, Excel Add-in, V1. https://www.gene-quantification.de/bestkeeper.html. Accessed 1 July 2019.

The NormFinder algorithm calculates a stability value for individual genes based on the inter- and intra-group expression variation of the candidate reference genes. The NormFinder algorithm is further able to select reference genes for a normalization factor based on their intergroup expression variation. The optimal number of genes is reached when the addition of a further gene does not lead to a reduction of the stability value. NormFinder software, Excel Add-in, V0.953. https://moma.dk/normfinder-software. Accessed 1 July 2019.

The GeNorm algorithm included in the Qbaseplus software is based on the analysis of a stability value for individual genes using a pairwise comparison approach across the whole sample set. GeNorm uses the most stable genes for building a normalization factor. The optimum number of reference genes to be used is calculated by pairwise variation analysis (Vn/Vn + 1) between two sequential normalization factors (NFn and NFn + 1). Typically, a variation value (V) < 0.15 is thought to predict sufficient expression stability. Qbase + software, V2.3, Biogazelle. http://qbaseplus.com. Accessed 1 July 2019.

### RNA-seq analysis

Systematic analysis of RNA-seq data for potential reference genes was performed as follows: Upper-quartile normalized read counts (UQ) were filtered using previously published criteria: (i) mean [log2(UQ)] > 5, (ii) standard-deviation (SD) [log2(UQ)] < 1, (iii) no sample log2(UQ) differing from mean [log2(UQ)] ≥ 2 [16, 17]. Candidate reference genes were ranked based on their coefficient of variation (CV = SD/mean).

Two different, previously published RNA-seq datasets were analyzed: (1) RNA-seq data from cord blood of preterm and full-term neonates [15]. Group characteristics: Gestational age (mean (range), weeks) 29.9 (24.7–34.1), n = 8 for the group of preterm and 38.9 (37.0–40.6), *n* = 8 for the group of full-term neonates. (2) RNA-seq data from peripheral blood of full-term neonates taken at day of life (DOL) 0 and additionally at either DOL 1, 3 or 7 [17]. This data is publicly available from NCBI Gene Expression Omnibus (https://www.ncbi.nlm.nih.gov/geo/). Both available datasets (GSE123070 and GSE111404) were merged to the combined dataset (n = 50 for DOL 0, n = 16 for DOL 1, *n* = 18 for DOL 3, *n* = 16 for DOL 7; 18 females (36 %), 32 males (64 %)) in order to analyze relative gene expression. For systematic analysis of this RNA-seq data both datasets GSE123070 (referred to as dataset “A”) and GSE111404 (referred to as dataset “B”) were analyzed independently. Subsequently, the intersection was re-analyzed against the combined dataset (dataset A + B).

### Statistical analysis

Maternal and infant characteristics were analyzed using a two-tailed t-test for metric variables or the Chi-square test for categorical variables. Non-normalized or normalized gene expression was calculated from ct values using the 2^−Δct^ or 2^−ΔΔct^ method. Relative gene expression was normalized to the mean of the preterm neonates (group 1) unless stated otherwise. Statistical analysis of gene expression was performed using 2-way ANOVA. All statistical tests were performed using GraphPad Prism V7.0.

## Supplementary Information

**Additional file 1:** **Figure S1** Birth mode does not influence candidate reference gene expression. Samples were grouped based on birth mode (a, b) or based on birth mode and gestational age (c, d). a Relative gene expression (normalized to the group mean of SVD) of each candidate reference gene or normalization factor. b Maternal and infant characteristics by group. c Relative gene expression (normalized to the group mean of C/S Preterm) of each candidate reference gene or normalization factor. d Maternal and infant characteristics by group. SVD, spontaneous vaginal delivery; C/S, caesarean section. a, c Data is presented as mean ± SD.

## Data Availability

The datasets used and/or analyzed during the current study are available from the corresponding author on request.

## Abbreviations

C/S: Caesarean section
CT: Cycle threshold
CV: Coefficient of variation
DOL: Day of life
HELLP: Hemolysis, elevated liver enzymes, low platelet count syndrome
IQR: Inter-quartile-range
RNA-seq: RNA sequencing
RT-qPCR: Reverse transcription quantitative polymerase chain reaction
SD: Standard deviation
SVD: Spontaneous vaginal delivery
UQ: Upper-quartile normalized read counts
