# Supplementary figures and images for: Validation of reference genes for whole blood gene expression analysis in cord blood of preterm and full-term neonates and peripheral blood of healthy adults
Source: BMC Genomics. 2021 Jun 30;22:489. doi: 10.1186/s12864-021-07801-0 (PMC8244134; doi:10.1186/s12864-021-07801-0)

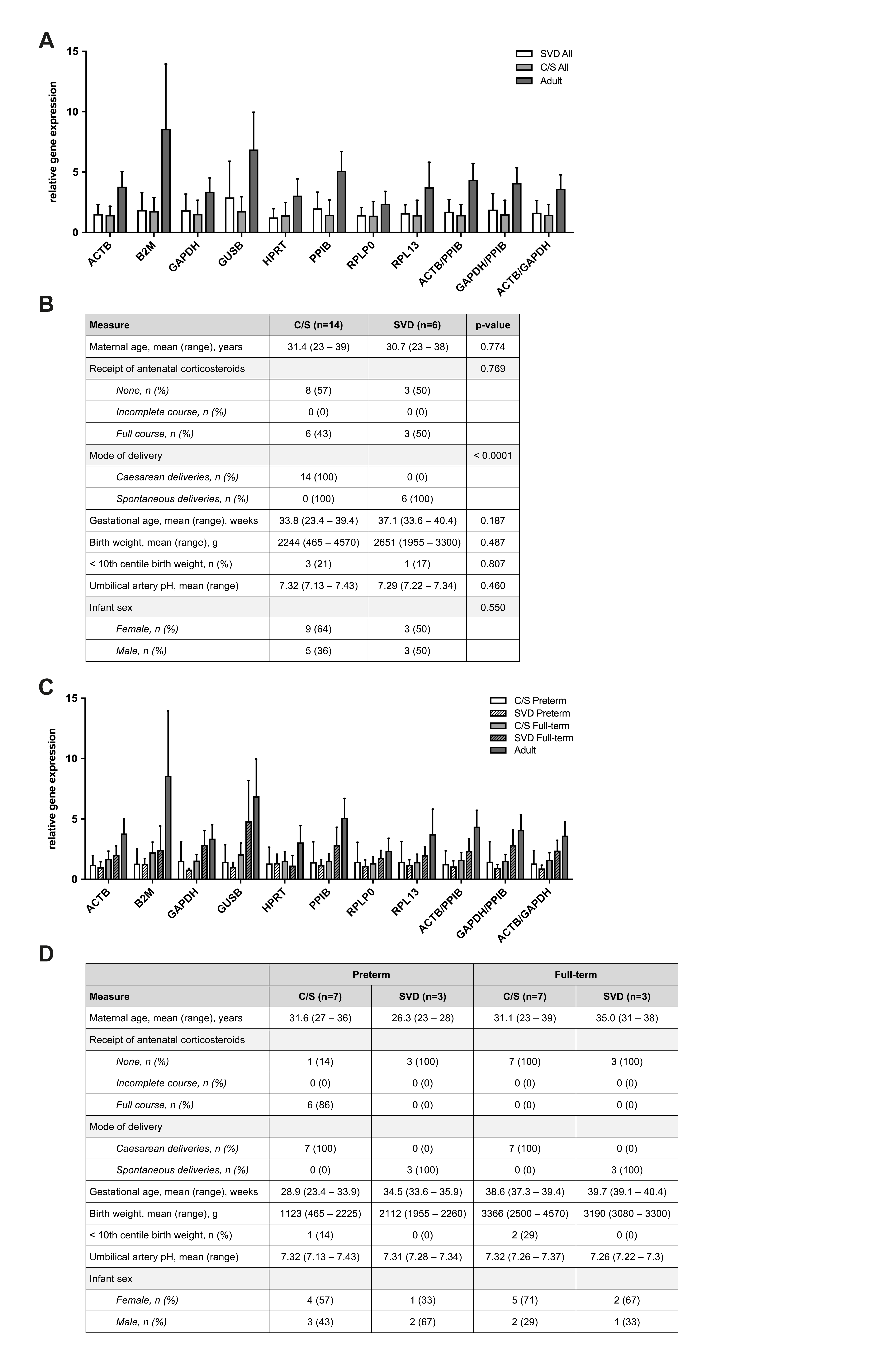

Supplement: Supplementary file 1 — Additional file 1: Figure S1 Birth mode does not influence candidate reference gene expression. Samples were grouped based on birth mode (a, b) or based on birth mode and gestational age (c, d). a Relative gene expression (normalized to the group mean of SVD) of each candidate reference gene or normalization factor. b Maternal and infant characteristics by group. c Relative gene expression (normalized to the group mean of C/S Preterm) of each candidate reference gene or normalization factor. d Maternal and infant characteristics by group. SVD, spontaneous vaginal delivery; C/S, caesarean section. a, c Data is presented as mean ± SD. [file 12864_2021_7801_MOESM1_ESM.tif]
